# Dystrophie cornéenne: Dystrophie granulaire ou de Groenouw Type 1

**DOI:** 10.11604/pamj.2014.17.74.3832

**Published:** 2014-01-31

**Authors:** Salim Belhassan, Rajae Daoudi

**Affiliations:** 1 Université Mohammed V Souissi, Service d'Ophtalmologie A de l'Hôpital des Spécialités, Centre Hospitalier Universitaire, Rabat, Maroc

**Keywords:** Dystrophie cornéenne, dystrophie granulaire, dystrophie Groenouw, oeil, acuité visuelle, corneal dystrophy, granular dystrophy, Groenouw dystrophy, eye, acuité visuelle

## Image en medicine

La patiente présente dans ce cas est âgée de 26 ans, ayant comme antécédent une consanguinité parentale de premier degré qui accuse une baisse de l'acuité visuelle progressive depuis l'age de 20 ans, associée à une douleur oculaire périodique sans facteurs déclenchant au niveau des deux yeux. L'examen clinique de la patiente trouve une acuité visuelle effondrée à compte les doigts de près à l'oeil droit et à compte les doigts à un mètre à l'oeil gauche. Au niveau du segment antérieur on trouve de manière bilatérale et quasi symétrique, des lésions cornéennes très centrales, diffuses, dispersées, non confluentes et respectant la périphérie, de couleur gris blanchâtre peu profondes siégeant au niveau du stroma cornéen; c'est la dystrophie de Groenouw Type 1. Il s'agit d'une dystrophie héréditaire transmise sur un mode autosomique dominant. En microscopie optique, on note l'existence de dépôts granuleux dispersés dans tout le stroma et sont plus concentrés dans le stroma antérieur. Dans certains cas la couche de Bowman et la membrane basale de l’épithélium peuvent être atteintes. En microscopie électronique, ces structures granuleuses correspondent à des corps en bâtonnets, denses aux électrons. Quand l'atteinte est superficielle le traitement de choix est la photokératectomie. Dans la forme plus profonde, l'indication de kératectomie transfixiante se pose, mais malheureusement l'affection récidive sur le greffon et nécessitera par la suite un traitement au laser.

**Figure 1:**
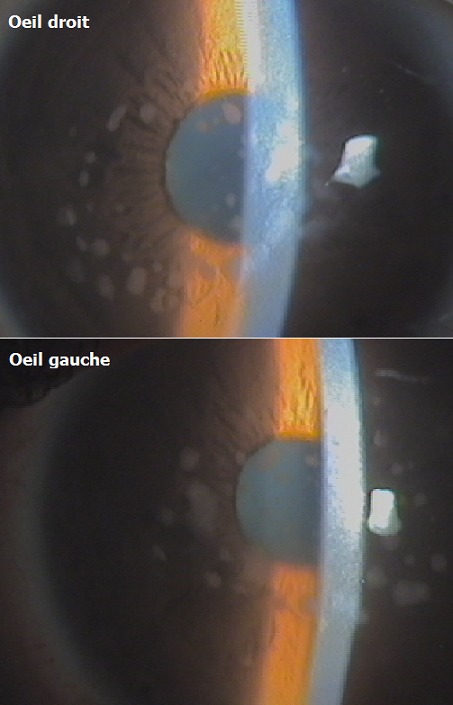
Montrant des lésions gris blanchâtres centrales retrouvées au niveau des deux yeux très caractéristiques de la dystrophie cornéenne de Groenouw

